# Survey of Network-Based Approaches to Research of Cardiovascular Diseases

**DOI:** 10.1155/2014/527029

**Published:** 2014-03-20

**Authors:** Anida Sarajlić, Nataša Pržulj

**Affiliations:** Department of Computing, Imperial College London, 180 Queen's Gate, South Kensington Campus, London SW72AZ, UK

## Abstract

Cardiovascular diseases (CVDs) are the leading health problem worldwide. Investigating causes and mechanisms of CVDs calls for an integrative approach that would take into account its complex etiology. Biological networks generated from available data on biomolecular interactions are an excellent platform for understanding interconnectedness of all processes within a living cell, including processes that underlie diseases. Consequently, topology of biological networks has successfully been used for identifying genes, pathways, and modules that govern molecular actions underlying various complex diseases. Here, we review approaches that explore and use relationships between topological properties of biological networks and mechanisms underlying CVDs.

## 1. Introduction

Cardiovascular diseases (CVDs) cover a broad range of disorders which affect different parts of cardiovascular system and include coronary diseases, carotid diseases, peripheral arterial diseases, and aneurysms. They remain the leading health problem which affects more than 80 million individuals in the United States alone [1]. Based on the data from 2009, in the United States, on average one person dies of CVDs every 40 seconds. Coronary heart disease alone causes one out of every six deaths [1]. By year 2020 it is expected that Brazil, Russia, India, and China will contribute significantly to a global increase of additional 4% of deaths caused by CVDs [2].

Etiology of cardiovascular diseases is not simple. There are forms of CVDs that are Mendelian disorders resulting from a mutation on a single gene [3]. However, the majority are complex diseases occurring as a result of an interplay between multiple genes [3], as well as a variety of factors such as diet, dyslipidemia, hypertension, and body mass index [4]. For addressing this complexity, an integrative approach, that would take into account coaction between the multiple causes behind CVDs, seems to be the method of choice. This is because properties of a complex system as a whole cannot be completely discovered by simply observing properties of individual parts of the system without taking into account their interconnectedness [5]. Hence, different systems biology approaches have been used in CVD research, which has recently been reviewed elsewhere [6–9].

A mathematical concept of a* network* has been introduced in systems biology as it accurately captures the inner workings of many complex biological systems. For example, metabolic pathways are interconnected into a network, providing redundancy, adaptability, and robustness [10], thus enabling energy-efficient production of metabolites. Also, the fact that a specific network topology comes as a direct consequence of biological processes occurring between the elements of the underlying system highlights the importance of the topology as a valuable source of new biological knowledge.

In this survey, we focus on network-based systems biology approaches to CVD research. More specifically, we aim to investigate the extent to which network topology has contributed to novel medical insights into CVDs.

## 2. Topology of Biological Networks Reveals Disease Genes, Modules, and Pathways

### 2.1. Biological Data and Networks

Recent advances in high-throughput techniques have resulted in a number of large-scale biological data sets. In Table 1, we list commonly used databases of molecular interaction and disease ontology data for* H. sapiens*. These databases accumulate biological information, including interactions and relationships among biological macromolecules and metabolites, such as protein-protein interactions (PPI), genetic interactions, or enzyme-substrate relationships. The available data also include gene functional annotations, pathway maps, information on genetic disorders, and disease associations. As an example of the scale of available data, BioGRID currently lists 303,268 nonredundant physical interactions between 51,129 proteins across 48 organisms, while DRYGIN (http://drygin.ccbr.utoronto.ca/) contains 5,482,948 genetic interactions for* S. cerevisiae*.

A network is the same as a mathematical concept of a graph, denoted as a pair *G* = {*V*, *E*}, where *V* is a set of vertices (nodes) and *E* is a set of links (edges) that connect pairs of nodes [11]. When constructing a graph it is necessary to determine how biological elements and relations between them correspond to nodes and edges. For example, an edge in a protein network can be placed between two proteins if they bind together to perform their biological function; this results in a commonly used protein-protein interaction (PPI) network. Conversely, an edge between two proteins can also be placed if the two proteins share a common trait, such as being targeted by the same drug or causing the same disease. These associations are usually found by mining the scientific literature, resulting in an association network. Other highly exploited networks are genetic interaction networks, where genes correspond to nodes in the graph and edges represent functional associations between genes: an interaction between two genes occurs when the result of mutations in the genes is not just a combination of phenotypes of those mutations [12]. A metabolic network is a union of all metabolic pathways within a cell, where nodes correspond to metabolites and enzymes, and directed edges are metabolic reactions [10, 13, 14]. Regulator-gene interactions can be summed up into a transcriptional regulatory network [15]. Given various experimental limitations, up till date, only a handful of transcriptional regulatory networks for complex biological systems have been defined [16].

Graph theoretic approaches offer insight into the structure of these networks and allow us to single out properties of a network, or its parts, which are different from expected by random. Such findings can reveal the connection between a specific topological characteristic and related biological function or a process, such as a disease. Here, we will not provide details on global and local network properties nor specific algorithms commonly used in graph theory, such as algorithms for network clustering or alignment. For more details on these topics, see [17–20].

Note that a limiting factor regarding network analyses is the quality of data. Although large amounts of biological data are available, they are still noisy and incomplete. Techniques used for obtaining the data are often biased—they may not provide enough sensitivity to detect all changes in the system [21]. Outcomes of experiments depend not only on experimental design but also on the stringency of conditions of the experiments: for example, too stringent conditions can lead to false negative interactions, as opposed to false positive results obtained from experiments that were not stringent enough. Also, depending on the focus of the research and experimental design, some genes/proteins can be favoured and their possible interactions are explored more often, such as those of disease genes. This can impose a particular structure in the network, for example, false hubs, without reflecting the underlying network topology. In addition, not all biological processes can be accurately represented as interactions (edges in the network) between two elements. Often a process in a biological system requires more than two elements and involves different types of interactions. However, a benefit is that network representation gives an opportunity to reduce the complexity of biological data that is required for performing computational analyses. Different data sources offer various insights into underlying biological processes, and, only if integrated, they will yield the full meaning. Network analysis provides exactly insight into interconnectedness of the data that describe different processes within a living cell. Below we give a short overview of methods that use biological networks to extract new knowledge about diseases. Specifically, we focus on network biology in CVDs.

### 2.2. Exploring Disease through Network Topology

**Figure 1:**
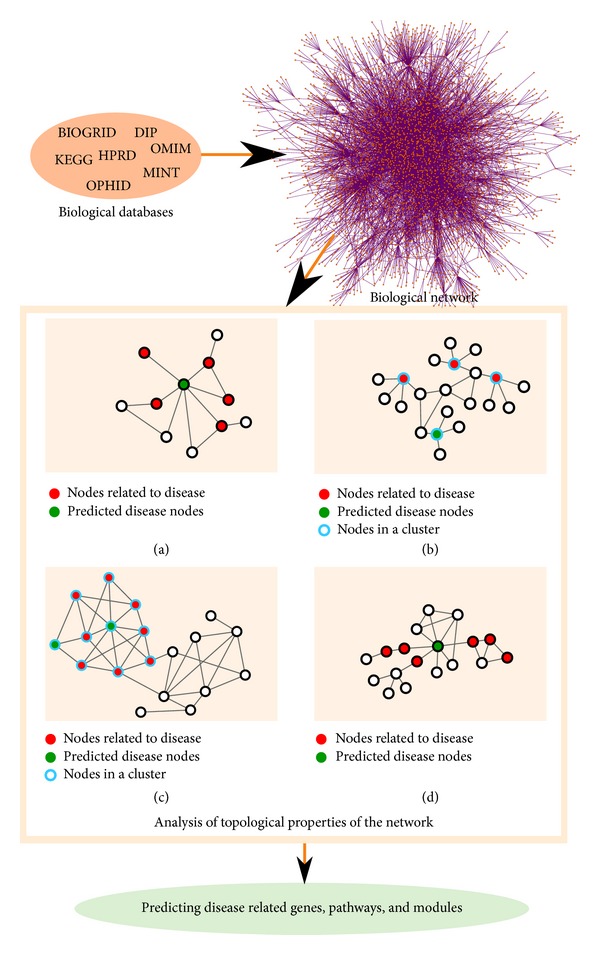
Using network topology to infer elements involved in disease. Panel (a): green node is associated with disease based on its neighbouring disease nodes (shown in red). Panel (b): nodes bordered in blue are part of the same cluster based on similar topology around them. Green node is associated with disease based on the cluster's enrichment in disease nodes (shown in red). Panel (c): nodes bordered in blue are part of the same graph cluster or community, in the network. Green nodes are associated with disease based on the community's enrichment in disease nodes (shown in red). Panel (d): node shown in green is associated with the disease, as a common node on shortest paths between nodes related to disease (shown in red).

Topology of PPI networks has widely been explored and used for inferring involvement of proteins in biological functions and processes, including diseases. It has been shown that proteins that are closer in the network are more likely to perform the same function [22]. In particular,* association by guilt* approach was used to infer functions of unannotated proteins: the direct neighbourhoods of proteins were examined looking for most common functions among annotated direct neighbours [23]. Similarly, the n neighbourhood of proteins [24] and shared neighbours of proteins [25] were analysed to annotate functions of unannotated proteins. These properties were used to associate genes with diseases using linkage methods (nomenclature adopted from [26]). In that sense, it has been shown that directly linked proteins in the human PPI network are more likely to cause similar diseases [27, 28] (simplified concept illustrated in Figure 1, panel (a)). A variant of linkage method was successfully applied to discover genes related to Alzheimer's disease [29].

Several other methods have shown that PPI network topology around proteins is a predictor of their function or their involvement in disease [30–32]. The local topology around a protein in a PPI network was summarized into a topological “signature” of a protein,* graphlet degree vector (GDV)* [30]. Proteins in the PPI network were grouped based on similarity of their “signatures,” or GDV similarity, and it has been shown that proteins within those groups belong to same protein complexes, perform the same biological function, and are part of the same subcellular components [30]. Also, GDV similarity between proteins in the PPI network was used as a similarity measure for clustering proteins using series of clustering methods, resulting in clusters significantly enriched in cancer and disease related proteins. This leads to predictions of new melanogenesis related genes purely from the topology of the human PPI network and the predictions were phenotypically validated [31, 32].

Described methods used clustering of nodes in the network based on their topological properties (simplified example is illustrated in Figure 1, panel (b)). Note that this is different from clustering the network by identifying its topological modules: locally dense neighbourhoods in the network called graph clusters or network communities [17] (Figure 1, panel (c)). It is generally accepted that a subset of nodes is a good cluster, or community, if the induced subgraph is dense, with relatively few connections between the cluster nodes and nodes that are in the remaining part of the graph [33]. These topological modules often correspond to* functional modules*: aggregations of nodes similar in function, and to* disease modules*: sets of nodes that contribute to a specific disease phenotype [26]. Mitra et al. [34] thoroughly reviewed integrative approaches for identifying such functional modular structures in biological networks. Accordingly, module-based methods use assumption that nodes belonging to same topological or functional module are highly likely to be involved in the same disease. These methods have often been applied in studies related to cancer [35–37]. Another example of this principle is modules identified using community discovery algorithm [38], which resulted in the discovery of new links between Alzheimer's disease and CVDs at the coexpression and coregulation levels [39]. Several module-based methods have been applied to research of CVDs, which will be elaborated in more detail later in this survey.

An interesting survey on different methods that use network topology for predictions of disease genes [40] pointed out that many of the methods that rely on clustering algorithms, or linkage-based inference, are outperformed by random walk-based methods. Random walkers diffuse along the network starting from disease involved nodes with the same probability of visiting any neighbouring node—most visited genes are considered to be on the disease pathway and potentially involved in a particular disease. A method for prioritization of candidate disease genes using random walk analysis was tested on 110 disease gene families and significantly outperformed methods based on distance measures such as linkage-based methods or methods based on shortest paths to disease proteins [41].

### 2.3. Disease Networks

We are currently witnessing an increase in using disease networks, networks of biomolecules involved in a particular disease or a group of diseases, for exploring relationships between different diseases. For example, Goh et al. [42] created a bipartite “diseasome” network, where one partition consists of a set of diseases and the other of a set of disease genes (and where, by definition of a bipartite network, all edges in the network are between the partitions). They used it to generate two network projections: disease gene network and human disease network (which they found is clustered according to major disorder classes). By exploring centrality and peripherality of genes in the gene network, they showed that contrary to essential human genes that encode hub proteins—highly linked proteins in network, the majority of disease genes do not encode hubs and are localized in the periphery of the network [42].

Janji$\overset{´}{\text{c}}$ and Pržulj [43] demonstrated the existence of topologically and functionally homogeneous “core subnetwork” of the human PPI network, which is enriched in disease genes, drug targets, and a small number of genes that have theoretically been proposed to be required for tumour formation, referred to as “driver genes” [44]. They call this subnetwork the “Core Diseasome” [43] and postulate it is the key to disease onset and progression and hence should be the primary object of therapeutic intervention.

CVD networks have recently gained interest, serving as a basis for a better understanding of the complexity behind the disease [6, 7]. In the next section we focus on various CVD networks with emphasis on the use of network topology. Note that henceforth we will use terms gene and protein interchangeably, as topological properties of proteins, represented as nodes the in PPI network, are commonly used to gain new knowledge about genes that encode these proteins.

## 3. Using Biological Networks in Research of CVDs

### 3.1. CVD Networks

There were several attempts to create biological networks relevant to various cardiovascular disorders.

A combination of methods based on experimental cell culture and data mining was used to collect a comprehensive set of vascular and atherosclerosis related genes [45]. In particular, public databases such as PubMed (http://www.ncbi.nlm.nih.gov/pubmed) were searched for genes related to the terms* atherosclerosis, smooth muscle cell, endothelial cell, apoptosis, cytokine, and adhesion molecule*. This list of genes was then combined with genes obtained from sequencing clones from stimulated vascular cells in culture. Next, a large association network was constructed through semantic mining of published literature—an association between two genes was extracted from sentences in scientific literature that contained two gene names and a verb as defined by user context file. Also, coronary artery segments isolated from explanted hearts of 22 cardiac transplant patients were experimentally processed, resulting in significant gene expression profiles obtained using significance analysis of microarrays (SAM) [46]. Then, for each gene from the large association network, a subnetwork was constructed. The subnetwork consisted of that gene and its neighbouring genes which were obtained using SAM analysis. A cumulative and average SAM scores were computed for each subnetwork and were used to identify subnetworks of high overall significance. Their central, “nexus,” genes were singled out as potential regulators that may cause the disease phenotype [45].

A similar method was used for constructing an association network of human in-stent restenosis [47]. Genes relevant to the disease were collected using methods based on experimental cell culture and data mining, while associations between genes were obtained through text mining of MEDLINE (http://www.nlm.nih.gov/pubs/factsheets/medline.html) abstracts. Again, a subnetwork for each gene was constructed containing the gene and its direct neighbours in the network. Gene expressions were experimentally assessed from tissue samples of 89 patients using SAM analysis. Subnetworks were next compared based on the overall significance score calculated using SAM scores of the subnetwork members. Central nodes of these subnetworks were identified as successful targets for drug therapy.

Skogsberg et al. [48] revealed a regulatory gene network of cholesterol-responsive atherosclerosis genes that control formation of plaques in arteries, using analysis of gene expression in response to plasma cholesterol-lowering. They established a list of genes related to atherosclerosis, foam cells, smooth muscle cells, endothelial cells, and T cells using automated text mining of PubMed abstracts. The resulting network was proposed as a starting point for future research of novel atherosclerosis therapies.

Another PPI network of cardiovascular diseases was created from CVD related proteins that were identified using protein annotations from Uniprot database (searching for the keyword* cardiovascular*) and known protein-protein interactions from HPRD [49]. Only proteins with at least one known interaction in HPRD were taken into account. In addition to these proteins, their interacting partners in the PPI network, which also appear in the signalling pathways from KEGG database, were included in the network. The resulting CVD PPI network consisted of 55 proteins and 122 PPIs and was used to identify network CVD biomarkers as follows. (1) Single biomarker discovery was based on significantly different expressions between proteins in control patients and disease patients (significantly low *P* values); biomarkers were evaluated using not only *P* values but also support vector machine (SVM). (2) A candidate* pair biomarker* is composed of two single biomarkers and a PPI between them. Pair biomarkers were selected based on the best performance in SVM and significantly low *P* values. (3) Candidate* triple biomarker* is composed of three single biomarkers and PPIs between every pair among them. Again, triple biomarkers were selected based on the best performance in SVM and significantly low *P* values. (4) Multiple CVD biomarkers were identified in similar manner as combinations of different single ones, pair ones, and triple biomarkers.

As mentioned in Section 2.1, despite their important biological role, human transcriptional regulatory networks are still largely unexplored. Some of the reasons are experimental limitations and human cellular diversity [16]. However, there have been several attempts to construct a cardiac transcription network. For example, mRNA profiles were integrated with DNA-binding events of key cardiac transcription factors (TFs) [50]. Insights into combinatorial regulation by cardiac TFs showed that they compensate each other's functions. Cardiac transcription network was built based on findings from RNA knockdown experiments. Target genes that are important for the cardiovascular system were chosen based on their biological roles such as muscle contractility and cardiac growth. The network depicted the common regulation of several transcriptional factors and the impact of the posttranscriptional modulation of expression levels by miRNAs [50]. Another transcriptional network of cardiac TFs and genes important for cardiac function was constructed based on coexpression analysis involving TFs critical for hearth development. Coregulatory relationships between five such TFs were revealed [51]. These types of relationships can give a new perspective for understanding the complexity of CVDs.

The quality of biological data is crucial for constructing a reliable CVD network, as discussed in Section 2.1. New technologies, such as next generation sequencing platforms, have significantly increased DNA sequencing output [52] and as such will largely increase the size of available biological data. Therefore, next generation sequencing methods for gene expression profiling will change the approaches to studying many common complex disorders, including CVDs [53]. The resulting new insights into underlying mechanisms of CVDs will yield more complete CVD networks and open a window of opportunities for exploring the topology of these networks.

### 3.2. Correlating Network Topology with CVD Mechanisms

Several authors tried to explore whether basic topological information from a biological network, such as connectivity of the nodes, can be correlated with biological properties required for CVD onset and progression.

One example is a global PPI network in heart failure (HF) [54], created as a subnetwork of PPIs from HPRD that includes HF relevant genes. Next, differentially expressed genes in HF were identified from microarray data encoding molecular profiles of healthy versus HF subjects. Proteins encoded by these significantly differentially expressed genes were also included in the HF PPI subnetwork. This network was used to explore the relationship between gene coexpression levels and their connectivity in the HF network. It was discovered that hub proteins in the network are encoded by genes that display a significant diversity of coexpression patterns in comparison to peripheral proteins. However, hub proteins are not necessarily encoded by genes that are significantly differentially expressed. Analysis of gene ontology (GO) terms [55] revealed the relationship between connectivity of the proteins in this network and their involvement in specific biological processes, such as processes related to cardiac remodelling.

In their later work, the same authors explored dilated cardiomyopathy (DCM) genes [56], as DCM is recognised as a leading cause of HF. DCM genes were identified using gene expression profiles from three independent datasets, while associations with HF were identified using literature mining. Human HF PPI network was created using PPIs from HPRD by including genes known to be involved in HF and genes from the gene expression datasets along with biological pathways associated with them. Again, connectivities of nodes (proteins) in HF PPI network were compared to their gene expression patterns. Differential gene expression was measured using SAM analysis, resulting in* di*values representing genes' score of class differentiation. Focusing on significantly differentially expressed genes, it was found that superhubs and hubs in the network had a lower range of* di*values, while genes that encoded peripheral proteins in the network had a higher range of* di*values.

Several module-based approaches were applied to various CVD networks attempting to identify functional modules related to the disease or discover new associations between genes and disease. Diez et al. [57] created a combined gene association and correlation network, using data from 47 microarrays from a database of carotid endarterectomies (Biobank of Karolinska Endarterectomies, BiKE (http://ki.se/start)). The gene correlation network was constructed using statistical analysis of gene expression data. The association network was constructed using the list of differentially expressed genes, by performing literature search for each gene symbol and association keywords such as “*gene A activates gene B.*” The networks were then merged into an undirected network of atherosclerosis. This network was searched for active modules based on closeness centrality using* jActiveModules* Cytoscape plugin [58]. APOC1 gene was differentially expressed in atherosclerotic plaque and related to several important GO categories characteristic of the disease mechanism, so it was selected for a more detailed analysis. Hence, among detected modules, the one containing APOC1 gene was further inspected. This module was checked for GO enrichment. GO categories relevant to atherosclerosis mechanisms and etiology that were identified in this module were all characteristic of APOC1 gene, suggesting its importance in this disease.

Ischemic dilated cardiomyopathy (ICM) is one of the main pathological forms of DCM. A set of genes differentially expressed in ICM, downloaded from gene expression omnibus (GEO) (http://www.ncbi.nlm.nih.gov/geo/), and cardiac myocytes proteins retrieved from human protein atlas (HPA) [59] were merged to create another CVD relevant PPI network [60]. Information about PPIs was integrated from several public databases. The analysed largest connected component of this PPI network was divided in four layers, based on subcellular localization information. This revealed that the extracellular and plasma membrane layers contained more downregulated genes, while cytoplasm and nucleus contained more upregulated genes. Next, significantly overrepresented biological processes (BPs) were identified, and PPI network containing only proteins related to these GO BPs was then divided into 12 clusters according to BPs. It was shown that the number of PPIs between proteins involved in different BPs was associated with differential gene expression patterns.

Rende et al. [61] used topological features of PPI networks in search of genes common to CVDs and other diseases, by identifying functional modules of genes. They extended a core CVD network, consisting of proteins known to be associated with CVDs (manually curated from the literature), by including their direct interactors in PPI network, resulting in a cardiovascular disease “functional linkage network” (CFN). Hub proteins in this network were considered to be the key nodes that regulate molecular mechanisms of CVDs and interdependence between CVDs and other complex disorders. These hubs were identified using distributions of node degrees and betweenness centralities. Functional modules, highly connected subgraphs, were identified using a modularity measure based solely on topological properties, allowing modules to overlap. All hub proteins appeared in these functional modules. Presence of a protein in multiple functional modules in addition to its high connectivity implied that any changes regarding protein would affect all its functional modules. Next, proteins in functional modules were matched to diseases from OMIM database: 19 modules were associated with CVDs. Also, modules associated with at least two diseases were examined for functional GO term enrichment and were shown to be functionally linked. This approach revealed some significant complex disorders that cooccur with CVDs and identified relevant shared disease genes and shared disease functional modules.

Known causal congenital heart disease (CHD) genes and genes differentially expressed in this disease (named* target genes*) were mapped onto a PPI network with the aim of identifying gene modules relevant to CHD [62]. The network was modelled as an electrical circuit, where edges between nodes (genes) were used as a conductance of a resistor according to correlation of coexpression between the two end nodes. Shortest paths from one causal gene to all target genes were merged into a subnetwork, and the current flow for each gene in the subnetwork was computed to evaluate its importance. Genes were assigned to a subnetwork in which they scored best. This resulted in 12 disjoint modules for further analyses: relationships of individual modules with disease phenotypes, mutual coexpression among genes within the modules, functional enrichment, and pathway analysis. As a result, candidate disease genes and hub modules that regulate key pathways of CHD were identified.

Functional modules of gene coexpression networks were also explored in research of cardiac development, hypertrophy, and failure [63]. Datasets from microarray experiments involving myocardial tissue were collected from GEO and used for creating a weighted gene coexpression network, where edges represent adjacencies between genes based on weighted Pearson correlation between gene expression profiles. Gene modules were identified using agglomerative hierarchical clustering of adjacencies given by the topological overlap measure based on shared network neighbours. The modules were first identified in fetal tissue, followed by evaluating their reproducibility in normal adult, hypertrophied, and failing myocardial tissue. The analysis revealed specific gene coexpression modules that were present both in developing heart and in hypertrophied or failing myocardial tissue.

### 3.3. Methods for Utilizing Network Topology in CVD Research

In previous section, we described a variety of methods that used biological networks in search of genes, pathways, or functional modules that are significant for different types of CVDs.

We see that the majority of approaches focused on constructing biological networks of particular cardiovascular disorders. Several approaches further explored topological properties of these networks and use them in search of new CVD knowledge. In particular, modules in the network of atherosclerosis [57] were identified based on closeness centrality. Functional modules of a CVD network used for investigating relationships between CVD and other disorders were identified using modularity measure based solely on network topology [61]. The method for identifying modules in CHD utilized shortest paths in the network between genes of interest [62]. Also, some basic topological properties, such as node connectivity [54, 56], or the number of interactions between functional sets [60], were examined in correlation with disease. Note that the vast majority of the above-presented topological analyses focused on CVD subnetworks in isolation, rather than observing them as parts of a larger, more complete interaction network, such as the entire human PPI network.

This may be a limiting factor when exploring the interplay between genes involved in different CVD disorders or when targeting genes that have previously not been connected to CVDs. The importance of observing the neighbourhood of disease genes in the entire PPI network was emphasized in one of the studies related to atherosclerosis [64]. Functional enrichment test performed only on differentially expressed genes failed to detect biological processes related to the disease progression. However, the network that included both differentially expressed genes and genes that have high connectivity with them in the entire PPI network was functionally enriched in relevant biological processes. This analysis showed that the regulators of disease progression should be looked for among genes that are not necessarily differentially expressed and within the context of the entire available PPI network.

We summarized the methods that used topology of biological networks in research of CVDs in Table 2. There are only few approaches that identified new genes relevant to CVDs relying solely on topological properties of entire PPI network. The example is the computational method based on six topological features (degree, neighbour count of disease genes, ratio of disease genes among neighbours, betweenness centrality, clustering coefficient, and mean shortest path length to disease gene) [65]. The constructed classifier was used on the PPI network to predict candidate genes for coronary artery disease.

The PPI network topology was also used for inferring proteins' involvement in CVDs as follows [66]. Proteins were clustered based on the similarity of topologies of their neighbourhoods in the PPI network, measured using* GDV similarity* [30]. The clusters were then checked for enrichment in CVD genes. The overlap of statistically significantly enriched clusters contained 10* key CVD genes* and 17 predicted new CVD related genes. More than 70% of these predictions were validated in the literature. Also, both* key CVD genes* and predicted CVD genes were enriched in biological functions that CVD drug mechanisms rely on, showing that this approach may be successful in identifying potential drug targets.

## 4. Conclusion

The emerging interest in molecular interaction networks of various cardiovascular diseases has resulted in a number of association, gene expression, PPI, and transcriptional regulatory networks being examined to study atherosclerosis, in-stent restenosis, heart failure, dilated cardiomyopathy, ischemic dilated cardiomyopathy, and CVDs in general. Many of these networks were constructed using experimental data combined with literature mining, with the aim of identifying a broader set of genes involved in a particular cardiovascular disorder. These networks are a valuable platform for exploring the mechanisms of the disease. Nevertheless, their topologies have not been fully explored.

We surveyed studies that explored the link between some basic topological properties of CVD genes in networks and involvement of these genes in specific disease related processes. Several CVD networks were checked for enrichment in biological functions relevant to the disease, and functional modules in the networks were identified, in some cases using topological properties. However, topological analysis was usually limited to the disease specific subnetwork, without observing it in the context of a larger, more complete network. Such complete interaction networks were analysed only in few studies, which explored the topology around genes that were previously not associated with CVD and thus not present in the disease specific subnetwork. This resulted in predictions of novel CVD genes.

There is a huge potential in analysing CVD related molecular subnetworks and their topology in the context of the complete biomolecular interaction networks. Such approaches could give better insight into interconnectedness of different CVDs. They could help discover novel CVD genes and pathways responsible for the dependency between different disorders.

## Figures and Tables

**Table 1:** Databases of human molecular interaction and disease ontology data.

Database name	Type of data	URL
BioGRID	PPI and genetic interactions	http://thebiogrid.org/
HPRD	PPI, disease associations, posttranslational modifications, tissue expression, subcellular localization, and enzyme/substrate relationships	http://www.hprd.org/
DIP	Experimentally determined PPI	http://dip.doe-mbi.ucla.edu/dip/
HomoMINT	PPI	http://mint.bio.uniroma2.it/HomoMINT/
OPHID	PPI	http://ophid.utoronto.ca/ophidv2.204/
KEGG	Pathway maps, human diseases, drugs, orthology groups, genes, relations within genes, metabolites, biochemical reactions, and enzymes	http://www.genome.jp/kegg/
OMIM	Information on genes and genetic disorders	http://www.ncbi.nlm.nih.gov/omim

**Table 2:** Methods that explored topology of biological networks in research of CVDs.

Network	Type of data/interactions in the network	Topological analysis performed on the data	Aims of topological analysis	Reference
Heart failure (HF) network	HF relevant genes, genes differentially expressed in HF and dilated cardiomyopathy (DCM), and PPI data	Connectivity of nodes	Relationship between gene connectivity and gene coexpression levels and their biological functions	[54, 56]

Network of atherosclerosis	Literature associations and gene expression data	Network modules identified based on closeness centrality	GO enrichment of network modules	[57]

Network of ischemic dilated cardiomyopathy (ICM)	Genes differentially expressed in ICM, cardiac myocytes proteins, and PPI data	Number of edges between network clusters	Correlation between number of edges between network clusters and differential gene expression patterns	[60]

Cardiovascular disease “functional linkage network” (CFN)	CVD proteins and PPI data	Degree distribution, betweenness centrality, and modularity measure	Associating functional modules (highly connected subgraphs) with diseases	[61]

Congenital heart disease (CHD) network	Known CHD genes, genes differentially expressed in CHD, and PPI data	Subnetworks based on shortest paths and current flow (network was modelled as an electrical circuit)	Functional subnetwork analysis in search of key pathways of CHD	[62]

Networks for analysis of cardiac development, hypertrophy, and failure	Gene coexpression data	Network modules based on hierarchical clustering and shared network neighbours	Identifying common modules in networks of different types of myocardial tissue	[63]

Human PPI network	PPI data	Node degree, neighbourhood enrichment, betweenness centrality, clustering coefficient, and shortest path length	Inferring coronary artery disease genes based on topological information	[65]

Human PPI network	PPI data	Clustering nodes based on graphlet degree vector similarity	Inferring new CVD genes based on clusters' enrichment in CVD genes	[66]
